# Promoting the accumulation of scopolamine and hyoscyamine in *Hyoscyamus niger* L. through EMS based mutagenesis

**DOI:** 10.1371/journal.pone.0231355

**Published:** 2020-05-21

**Authors:** Durdana Shah, Azra N. Kamili, Aijaz A. Wani, Umer Majeed, Zubair Ahmad Wani, Nasreena Sajjad, Parvaiz Ahmad

**Affiliations:** 1 Plant Tissue Culture Lab, Centre of Research for Development, University of Kashmir, Srinagar, J&K, India; 2 Cytogenetics and Reproductive Biology Lab, Department of Botany, University of Kashmir, Srinagar, J&K, India; 3 Immunology Lab, Department of Biotechnology, University of Kashmir, Srinagar, J&K, India; 4 Department of Biochemistry, University of Kashmir, Hazratbal, Jammu and Kashmir, India; 5 King Saud University, Riyadh, Saudi Arabia; 6 Department of Botany and Microbiology, College of Science, Srinagar, Jammu and Kashmir, India; National University of Kaohsiung, TAIWAN

## Abstract

The overexploitation of medicinal plants is depleting gene pool at an alarming rate. In this scenario inducing the genetic variability through targeted mutations could be beneficial in generating varieties with increased content of active compounds. The present study aimed to develop a reproducible protocol for *in vitro* multiplication and mutagenesis of *Hyoscyamus niger* targeting putrescine N-methyltransferase (PMT) and 6β-hydroxy hyoscyamine (H6H) genes of alkaloid biosynthetic pathway. *In vitro* raised callus were treated with different concentrations (0.01% - 0.1%) of Ethyl Methane Sulfonate (EMS). Emerging multiple shoots and roots were obtained on the MS media supplemented with cytokinins and auxins. Significant effects on morphological characteristics were observed following exposure to different concentrations of EMS. EMS at a concentration of 0.03% was seen to be effective in enhancing the average shoot and root number from 14.5±0.30 to 22.2 ±0.77 and 7.2±0.12 to 8.8±0.72, respectively. The lethal dose (LD_50)_ dose was calculated at 0.08% EMS. The results depicted that EMS has an intense effect on PMT and H6H gene expression and metabolite accumulation. The transcripts of PMT and H6H were significantly upregulated at 0.03–0.05% EMS compared to control. EMS treated explants showed increased accumulation of scopolamine (0.639 μg/g) and hyoscyamine (0.0344μg/g) compared to untreated.

## Introduction

Tropane alkaloid biosynthesis in *Hyoscyamus niger* begins with the methylation of putrescine to N-methylputrescine by putrescine N-methyltransferase (PMT) as putrescine is the common precursor of tropane alkaloids [1]. Scopolamine (6,7-β-epoxide of hyoscyamine) being the final product of this pathway, is formed from hyoscyamine using 6β-hydroxy hyoscyamine followed by intermolecular epoxide formation catalyzed by hyoscyamine 6β-hydroxylase (H6H) [2]. The cDNA of which has been cloned from several tropane alkaloid-containing plants such as *Atropa belladonna* [3], *Anisodus acutangulus* [4] *and Datura arborea* [5]. Therefore, H6H and PMT are promising target enzymes that, if overexpressed due to mutation, in hyoscyamine-accumulating tissues, would result in increased scopolamine and hyoscyamine levels in the variants. Plant alkaloids constitute the largest group of natural products providing many pharmacologically active compounds. Among tropane alkaloids, hyoscyamine and scopolamine are medicinally used in analgesic, sedative, antispasmodic and also have mydriatic properties [6]. Illnesses such as ear and eye inflammation, rheumatism, ulcers, cough, motion sickness, asthma, rabies, fevers, bronchitis, renal colic, and spasm are treated using this medicinal plant [7].

Nowadays several techniques are being used to increase the alkaloid content of the plants. One of the commonly used techniques is *in vitro* mutagenesis that induces stress tolerance, improves the yield and quality of crop plants and provides an opportunity to increase the alkaloid content of economically important cultivars. *In vitro* mutagenesis is an efficient, fast, and low-cost technique important for crop improvement [8]. Since genetic variability is required for the betterment and improvement of medicinal plants, that take place naturally through spontaneous mutation or could be induced by chemical or physical mutagens [9]. Nevertheless, the rate of spontaneous mutations is quite less and limits its use for the propagation of new cultivars [9]. Thus, variability could be prompted by the use of chemical and physical mutagens, T-DNA insertional mutagenesis and tissue culture-derived variation or somaclonal variation [9]. Chemical mutagenesis induces about 70–99% of the changes in a plant system and generally causes single base-pair (bp) alteration and single-nucleotide polymorphisms [10]. However, out of all the chemical mutagens, Ethyl Methane Sulfonate (EMS) is most widely used in plant systems. The mutagen alkylates guanine bases, triggering the DNA-polymerase to approve thymine insertion over cytosine opposite to O-6-ethyl guanine, and leads to the random point mutation [11]. *In vitro* cultures show genetic variation in terms of changes in phenotype, such as alteration in plant height and plant architecture, and the number of leaves and branches, pigmentation, yield potential [12]. The nucleotide variations are also seen at the gene level due to chemical mutagens [13]. To date, more than 3218 mutant varieties have been released worldwide as a result of induced mutations [14]. The application of biotechnological tools particularly a combination of *in vitro* method with chemical-induced mutagenesis suggests improvement of various cultivars to superior varieties [15]. These improvements provide an ideal approach for the propagation of medicinal plants with superior genotypes, that could be further released to normal habitats for cultivation on a large scale [16]. Molecular techniques are used in assessing plant genetic variability, gene expression and applied in the analysis of specific genes, as well as to understand gene action, gene expression and generate genetic maps, so on. In the present study, *Hyocyamus niger* was selected for *in-vitro* mutagenesis with special reference to PMT and H6H genes of secondary metabolite pathway to observe the effect of EMS on hyoscyamine and scopolamine. *Hyocyamus niger* is a commercially important plant for its rich repository of alkaloid production primarily hyoscyamine (HYO) and scopolamine (SCO) [17, 18]. These alkaloids have complex chemical structures, and industrial synthesis is excessively costly. Therefore, the plant resources mainly solanaceae plants are harvested at an alarming rate [19]. Thus, mass propagation and mutagenesis under *in vitro* conditions was undertaken for the improvement of this plant. However, various reports suggest mass propagation of *Hyocyamus niger* [19, 20], limited reports are available on *in vitro* mutagenesis of *Hyoscyamus niger* along with expressional and HPLC analysis. In current study, *in vitro* mutagenesis using EMS, sterilization, callus induction, shoot and root multiplication, acclimatization protocol was developed using different hormonal regimes. In addition, mutational analysis in PMT and H6H genes through gene sequencing and expressional and HPLC analysis was carried out.

## Material and methods

Plant samples were collected from Pahalgam area (34°13^′^ 30^′′^ N 75° 26′ 20′′ E) in south Kashmir during July 2017. Different parts of the plant such as leaves, stem, cotyledons, and hypocotyls were excised and used as explant. The explants were then washed with detergent (labolene and extran 0.5%) followed by the drops of surfactant (tween-20). After washing, the explants were subjected to chemical sterilization using ethanol (70–100%), sodium hypochlorite 5,10, 15 and 20% (v/v), and mercuric chloride (HgCl_2_) 0.01 and 0.02 (v/v) for different times periods. Finally, explants were cleaned carefully several times with autoclaved double distilled water.

### Media and culture conditions

Murashige and Skoog (MS) media [21] containing 30% sucrose was augmented with different range of cytokinins and auxins and was solidified using 8% Agar (Hi media). The pH was adjusted to 5.5 with 1N HCl or 1N NaOH and finally dispensed into 100ml Erlenmeyer flasks (borosilicate glass) plugged with non-absorbent cotton. The media was sterilized in an autoclave for 20 minutes at 121°C and after inoculation cultures were incubated at 25± 3°C under 16/8h (light/dark) photoperiod, illuminated with cool white fluorescent tubes (20 watts), at a photon flux rate of 100μmol m^-2^ S^-1^.

### Callus formation and biomass

Leaves, stem, cotyledons, and hypocotyl were used as explants and cultured on MS media augmented with BAP, BAP+NAA, Kn, Kn + NAA, Kn+2-4-D at different concentrations. Callus formation was obtained at 25±2°C in 16/8h (light/dark) of photoperiod under sterile conditions.

Callus was collected after 6 weeks of culture period and weighed using fresh and dry weight. The callus was weighted before and after incubation at 35°C for 3 days to record fresh and dry weight. Using formula callus biomass was recorded:

$$\frac{\mathrm{Fresh}\;\mathrm{weight}-\mathrm{Dry}\;\mathrm{Weight}}{\mathrm{Dry}\;\mathrm{Weight}}$$

### Shoot differentiation and root formation

*In vitro* grown callus was transferred on MS media enhanced with different hormonal concentrations (BAP+ NAA, BAP + IAA, BAP, TDZ, TDZ + NAA). Shoot differentiation and elongation were carried out under 25±3°C with 16h light/ 8 h dark photoperiod. Calli (6–8 mm) were transferred on MS medium augmented with TDZ (1–20μm), BAP (1–16μm), TDZ+IBA, TDZ+NAA, BAP+NAA, TDZ+IAA as well as sucrose 30g/l and agar 8gm/l.

Callus without shoots was transferred on MS media augmented with different hormonal concentrations of IBA, IAA, and NAA. After 8 weeks the successful root initiation was recorded on MS medium containing NAA (0.5μm-8μm), IBA (0.5–8μm) and IAA (0.5–8μm) individually.

### Hardening

*Ex vitro* culture was carried out in a greenhouse under 70% shading and 80% humidity. Rooted shoots were washed to remove medium and were planted in plastic pots containing sterilized sand, field soil and vermiculite (1:1:1).

### Ethyl Methane Sulfonate treatment

To see the effect of chemical mutagen (EMS) on callus biomass, morphological characters, expression of genes (PMT and H6H) and content of hyoscyamine and scopolamine of *Hyoscyamus niger* L., small pieces of calli weighing about 100 mg were treated with different concentrations of EMS. A stock solution (1% EMS) was prepared by dissolving 1ml EMS in 99ml of Aq. DMSO (70%). From this stock solution, different concentrations were calculated by using formula N1V1 = N2V2. Then, explants were exposed to mutagenic treatment (0.00, 0.01, 0.02, 0.03, 0.04, 0.05, 0.06, 0.70, 0.08, 0.09 and .1 v/v) % for 1 hr. Treated and non-treated calli were shifted to multiple shooting media containing TDZ (16 μM) +IBA (3.5 μM) under aseptic conditions. Rooting was induced from EMS treated calli, cultured on MS + IBA (8μM). The data was scored in terms of root and shoot development percentage, root and shoot number per explant, root and shoot length and callus biomass.

### DNA extraction and PCR analysis

For DNA isolation, leaf samples were collected from plants regenerated via EMS treated callus. The samples were crushed 3 to 5 times in liquid nitrogen. A 100mg of sample powder was transferred to a 2ml tight capped Tarsons tubes. Total genomic DNA was extracted from control and EMS treated samples using a DNA isolation kit (DNeasy- Plant Mini kit-Qiagen, Germany). The extracted DNA was quantified by Nanodrop ND-1000 spectrophotometer (Nanodrop Technologies, Wilmington, DE, USA). DNA integrity was checked on 1.0% (w/v) agarose gel using 1X TAE (Tris-acetate EDTA) as a running buffer.

Primers specific to PMT and H6H were constructed from the National Center for Biotechnology Information (NCBI database) to amplify both the full length (S1 Table) and partial sequences (Table 1). DNA samples from the control and the treatments were subjected to PCR analysis [22] targeting H6H and PMT gene for amplification. PCR amplifications were carried out in a reaction volume of 50μl containing 50–100 ng of genomic DNA, 0.2 mM of each dNTP, 0.1μM of each primer, and 0.01 units of Taq/μl DNA polymerase (Sigma) in 1x Taq Buffer containing Tris-HCl, KCl, (NH_4_)_2_SO_4_, 1.5mM MgCl_2_; pH 8.7. Amplification was done using PCR program as denaturation at 94^ο^C for 10 min, followed by denaturation for 30 seconds, annealing at 45°C (H6H gene) and 54°C (PMT gene), extension at 72°C for 1 minute and a final extension at 72°C for 5 minutes. Amplification products were analyzed on 1.5% agarose gel along with Ikb DNA ladder.

**Table 1:** Primer characteristics of PMT and H6H genes used for PCR analysis.

Primer Name	Direction	Sequence (5’ → 3’)	PCR product (bp)
PRIMER I (PMT)	Forward (SI)	AGAGCAGAGCCATAGGTTTCA	400
	Reverse (RI)	CTGGTACCTTGTTAAGAATGGGCA	
PRIMER II (H6H)	Forward (SII)	CTGGCCTGAAAAACCAGCAA	500
	Reverse (RII)	GTGGTGGGTTGTCTTGGTTGA	

Sequencing was done commercially using the services of SCIGENOM COCHIN, KERELA. DNA sequences of the amplicons were obtained in FASTA and pdf formats. The pdf file of each DNA sequence was used for visual inspection of the sequencing chromatogram using MEGA-X software.

### Expression analysis

RNA was isolated from leaf samples of *in vitro* grown treated callus and control samples using Trizol method (Invitrogen). Briefly, 0.5g of the leaf sample was taken for RNA extraction followed by thoroughly grinding the samples in liquid nitrogen until fine powder was obtained. The samples were then transferred into 1.5ml ice-chilled tubes and 1ml of Trizol was added to each tube and vortexed for 30–45 sec followed by incubation at room temperature for about 10–15 minutes. The tubes were then centrifuged at 12000rpm for 10 minutes at 4°C. The pellet was discarded and the supernatant was carefully decanted into a fresh 1.5ml tube. To the supernatant 2.5 volumes of 95% isopropanol plus 1/10 volumes of 3M sodium acetate was added and incubated at 4°C for 30 minutes followed by centrifugation at 12000rpm for 10 minutes. Then, the supernatant was discarded and the pellet was washed twice with 70% ethanol. Finally, the pellet was dried and resuspended in 40μl of RNase free water [23].

The isolated RNA was quantified using the Nanodrop ND-1000 spectrophotometer. Samples with 260/280 ratio in the range of 1.8–2.0 were retained for further analysis. The quality of RNA was assessed by loading 1μg of RNA mixed with an equal volume of RNA Gel Loading Dye 2X (Thermo Scientific) on 1.0% (w/v) formaldehyde agarose gel using 1x MOPS as running buffer at 3V/cm. RNA was amplified with an internal control **(**specific primers**)** to check DNA contamination (S2 Fig).

### DNase treatment and cDNA synthesis

2μg RNA from each sample was treated with DNase I, RNase-free (Fermentas, Pittsburgh, PA, USA) to remove DNA contamination. To synthesize cDNA, RNA was reverse transcribed with the help of enzyme reverse transcriptase, an RNA-dependent DNA polymerase. Together with deoxynucleotide, triphosphates, magnesium ions and at neutral pH, the reverse transcriptase synthesizes a complementary DNA on the mRNA template. Finally, 1 microgram of RNA was used for cDNA synthesis [24]. The cDNA quality was checked by performing a PCR using tubulin and gene-specific primers (S2 Fig). The cDNA thus generated was used for other downstream processes like qRT-PCR. The cDNA was stored at -20°C until further use. The quality of the cDNA was assessed by setting a normal PCR reaction using gene-specific primers (S2 Table).

### Quantitative real‑time PCR

Quantitative PCR (qPCR) was carried out on Light Cycler 480 (Roche) platform with DNA Master SYBR Green kit (Roche) as a dye to evaluate the fold change in the mRNA level of PMT and H6H using different templates obtained from different treatments and corresponding control The reaction mix was prepared with the following ingredients: 10μl SYBR Premix, 1.5 μl cDNA, 0.3 μl of each primer (forward and reverse), 8.2 μl dH2O, with final volume of 20 μl. qPCR was performed using 1μl (~50ng) aliquot of the diluted first-strand cDNA template in a final reaction volume of 10μl containing 5μl of 2X SYBR Green (Roche), 0.5μl (10 Picomole) of gene-specific primers and 3μl of RNase free water. As an internal control to normalize each cDNA sample for variation in the amount of template, a primer specific was used as an internal control (Tublin). To ensure the amplification of a single product with the expected melting temperature and the absence of primer dimers, a melting curve analysis was performed. The relative transcript abundance of each gene was calculated from C_t_ values using three technical replicates. The relative expression of PMT and H6H was calculated using ΔΔCT method [25].

### HPLC analysis

HPLC analysis was carried out on a Shimadzu chromatographic apparatus (Kyoto, Japan) using a Symmetry C_18_ column. The mobile phase was selected variably at a flow rate of 1 ml min−1. Hyoscyamine and scopolamine were obtained from Sigma-Aldrich and prepared in methanol (1mg/ml). Hairy roots were extracted from *in vitro* raised (treated and control) samples and oven-dried at 55°C for 16 hr followed by crushing in liquid N_2_. Then, 3g of the crushed sample was dissolved in 10 ml of 15:5:1 (Methanol: Ammonium Hydroxide: Chloroform), followed by extraction with CHCl_3_ (10 ml per sample) was carried out and sonicated of samples for 10 minutes. The samples were kept for 1-hour at room temperature and evaporated on the rotatory evaporator till dryness. Further, 3ml of chloroform and 1.5 ml of 1NH_2_SO_4_ was added to each sample and then air-dried to remove the residual CHCl_3_. The pellet was resuspended in 28% NH_4_OH (pH = 10). The suspension was then filtered using filter paper. The filtrate is collected in a tube and the filter paper was washed with 1–2 ml CHCl_3_ to purge the membrane of residual metabolites. The samples were again evaporated on the rotatory evaporator with the addition of 2 ml methanol. Further, using the corresponding standards, the samples were quantified and stored for further analysis [26].

### Statistical analysis

For all the experiments, three biological replicates were used and each experiment was repeated thrice. Results were evaluated as mean ±SD. The data collected was analyzed by One-way Analysis of Variance (ANOVA). Significant differences were calculated at *P<0.05; **P<0.01; *P<0.001 using multiple Tukey’s and Duncan’s multiple comparison tests.

### Ethics statement

For sample collection, no specific permissions were required for the location mentioned above. The location is an open area with free access to the public. Besides, the study did not involve any endangered or protected species.

## Results

### Sterilization

In the present study, sterilization was achieved by treating different explants (leaves, stem, cotyledons, and hypocotyls) with ethanol, sodium hypochlorite (NaClO) and mercury chloride (HgCl_2_) at different concentrations and periods. The 100% sterilization and 100% survival rate was obtained at 0.02% (w/v) HgCl_2_ for 25 minute (Table 2).

**Table 2:** Effect of different chemical sterilants on-field explant sterilization, growth pattern and survival rate of *H*. *niger* L.

Chemical sterilants (%)	Duration	Sterilization	Contamination	Explant survival	Growth
	(min)	(%)	(%)	(%)	pattern
Ethanol(70)	10	0	100	-	NG
Ethanol(70)	20	0	100	-	NG
Ethanol(70)	30	10	90	-	NG
Ethanol(100)	10	10	90	-	NG
Ethanol(100)	20	25	75	5	NG
Ethanol(100)	30	30	70	10	NG
NaClO(5)	10	0	100	-	NG
NaClO (10)	15	10	90	-	NG
NaClO (15)	15	15	85	15	NG
NaClO (20)	20	20	80	20	M
NaClO (20)	30	25	75	30	M
HgCl_2_(0.01)	15	10	90	20	M
HgCl_2_(0.01)	20	15	85	20	M
HgCl_2_(0.01)	25	30	70	40	M
HgCl_2_(0.01)	30	45	55	45	V
HgCl_2_(0.01)	35	60	40	55	V
HgCl_2_(0.02)	15	70	30	90	V
HgCl_2_(0.02)	20	95	5	100	V
HgCl_2_(0.02)	25	100	0	80	V
HgCl_2_(0.02)	30	100	0	75	M
HgCl_2_(0.02)	35	100	0	50	M

*Data scored after 4 weeks of the culture period (n = 10): NG, no growth; V, vigorous; M, morbid.

### Callusing and callus biomass yield

**Fig 1:**
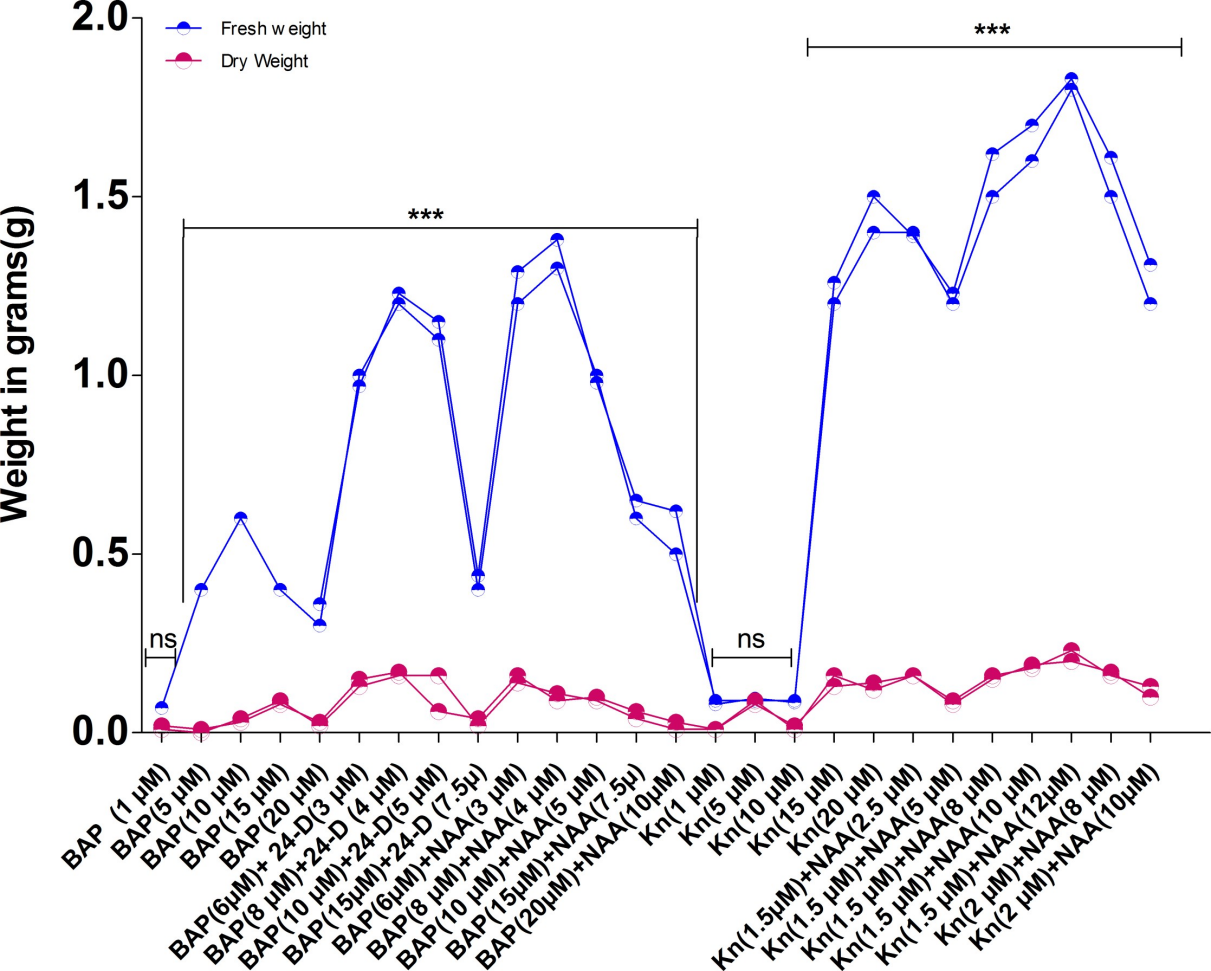
Effect of different concentrations of growth regulators on the fresh and dry weight of callus of *Hyoscyamus niger*. The samples were collected after 6 weeks of incubation period. The data represents the mean of three biological replicates. For each replicate, a total of 10 explants were used. An asterisk represents the significant differences between explants treated with different growth regulators at **P<0.05; **P<0.01; ***P<0.001.

**Fig 2:**
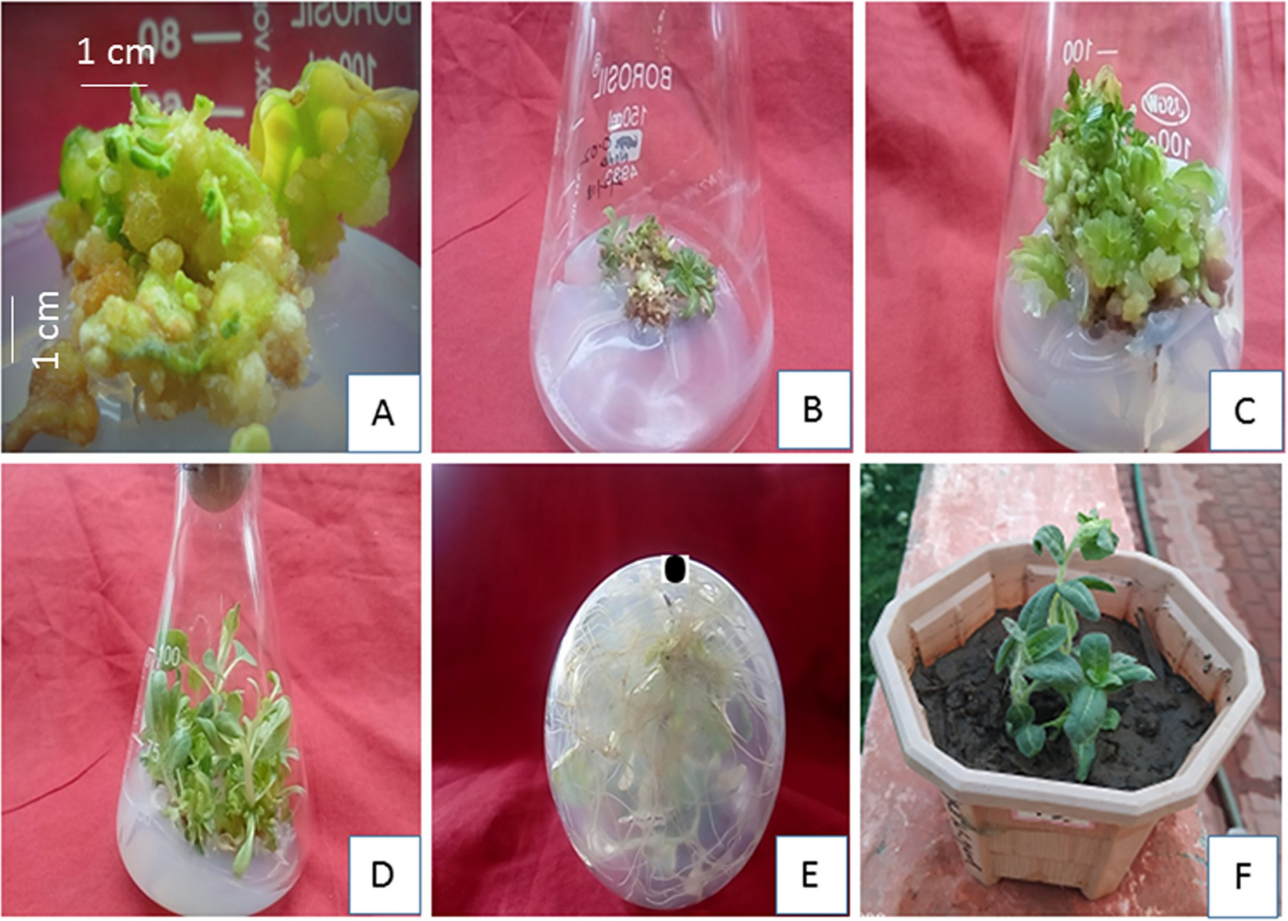
*In vitro* regeneration of *H*. *niger* from callus explants. **(**A) Callus formation, (B) Shoot incitation from callus explants (C) Shoot multiplication, (D) Shoot elongation, (E) Rooting (F) Hardening.

Growth hormones like cytokinins and auxins are used alone or in combinations to generate the maximum callus mass. Nevertheless, the best response for callus formation with Green Compact Nodular Callus (GCNC) was obtained from leaf explants followed by hypocotyl; no growth was recorded in case of stem and cotyledons. Best callus formation was recorded in both leaf and hypocotyl explants at Kn (1.5 μM) +NAA (12μM) with Green Compact Nodular Callus (GCNC) with 100% and 85% callusing percentage, respectively (Table 3, Fig 2A). Maximum callus biomass (1.8g/explant fresh weight) was obtained at Kn + NAA using leaf explants (Fig 1) as auxins initiated the best callus response and development.

**Table 3:** Effect of different hormonal treatments on callus formation of *Hyoscyamus niger* L. from field leaves and hypocotyl on MS media.

Treatments	Callusing (%)	Callus intensity	Callus nature	Callusing (%)	Callus intensity	Callus nature
	Leaf explants			Hypocotyl explants		
BAP (1 μM)	-	-	-	-	-	-
BAP(5 μM)	30	+	CCC	10	+	CCC
BAP(10 μM)	35	++	CCC	20	+	CCC
BAP(15 μM)	30	+	CCC	10	+	CCC
BAP(20 μM)	20	+	CCC	-	-	-
BAP(6μM)+ 2,4-D(3 μM)	65	++	LGCC	40	++	CCC
BAP(8 μM)+2,4-D (4 μM)	70	+++	LGCC	50	+	LGCC
BAP(10 μM)+2,4-D(5 μM)	55	++	LGCC	25	+	CCC
BAP(15μM)+2,4-D (7.5μ)	30	+	CCC	10	+	CCC
BAP(6μM)+NAA(3 μM)	75	+++	LGCC	50	++	CCC
BAP(8 μM)+NAA(4 μM)	80	+++	LGCC	60	++	LGCC
BAP(10 μM)+NAA(5 μM)	65	++	LGCC	35	+	LGCC
BAP(15μM)+NAA(7.5μ)	40	++	LGCC	25	+	CCC
BAP(20μM)+NAA(10μM)	30	++	LGCC	20	+	LGCC
Kn(1 μM)	-	-	-	-	-	-
Kn(5 μM)	-	-	-	-	-	-
Kn(10 μM)	-	-	-	-	-	-
Kn(15 μM)	30	+	CCC	20	+	CCC
Kn(20 μM)	20	+	CCC	10	+	CCC
Kn(1.5μM)+NAA(2.5 μM)	50	++	LGCC	35	++	LGCC
Kn(1.5 μM)+NAA(5 μM)	80	++	GCNC	65	++	LGCC
Kn(1.5 μM)+NAA(8 μM)	90	+++	GCNC	70	++	GCNC
Kn(1.5 μM)+NAA(10 μM)	95	+++	GCNC	75	+++	GCNC
Kn(1.5 μM)+NAA(12μM)	100	+++	GCNC	85	+++	GCNC
Kn(2 μM)+NAA(8 μM)	85	++	GCNC	65	++	LGCC
Kn(2 μM)+NAA(10μM)	70	++	LGCC	45	+	LGCC

* Data scored after 6 weeks of culture period (n = 10), (+) low callus; (++) moderate callus; (+++) intense callus, CCC (Compact Creamy Callus), LGCC (Light Green Compact Callus), GCNC (Green Compact Nodular Callus).

### Shoot/Root differentiation and Hardening

Shoot formation is desirable and necessary for every tissue culture protocol. In this regard *in vitro* raised callus was subcultured on MS media enhanced with different growth regulators at different concentrations for shoot proliferation. The study reinforced that MS + TDZ (14μM) + IBA (3.5μM) showed maximum shoot number (14.4±0.26) from callus explants (Table 4, Fig 2C and 2D). However, BAP was found to be less effective in shoot regeneration compared with TDZ.

**Table 4:** Effect of MS medium augmented with different concentrations of (BAP/TDZ/NAA/IAA/IBA) on shoot formation from *in vitro* raised callus of *H*. *niger* L.

Treatments	Shoot Number	Shoot Length(cm)	Shooting Response (%)
Control (MS basal)	-	-	-
BAP(1μM)	^a^3.6± 0.53	^a^1.6±0.58	93
BAP(4 μM)	^d^4.7± 0.60	^c^2.2±0.58	92
BAP(8 μM)	^f^6.7± 0.58	^c^2.2±0.75	100
BAP(12 μM)	^e^4.9± 0.67	^b^2.0±0.44	80
BAP(16 μM)	^b^4.0± 0.21	^b^2.0±0.54	77
TDZ (1 μM)	^g^7.8± 0.25	^d^2.4±0.46	97
TDZ (4 μM)	^h^8.2± 0.67	^e^2.6±0.85	98
TDZ (8 μM)	^j^8.8± 0.48	^f^2.8±0.33	100
TDZ (12 μM)	^k^9.9± 0.26	^g^3.1±0.24	100
TDZ (16 μM)	^o^13.3±0.10	^j^3.4±0.86	97
TDZ (20μM)	^l^10.0± 0.54	^c^2.2±0.26	90
BAP(3 μM)+NAA(1 μM)	^bc^4.2±0.56	^e^2.6±0.46	90
BAP(4 μM)+NAA(1 μM)	^d^4.6±0.67	^c^2.2±0.25	96
BAP(5 μM)+NAA(1 μM)	^bc^4.2±0.23	^f^2.8±0.23	90
TDZ (1 μM) +NAA(1 μM)	^h^8.0±0.45	^f^2.8±0.35	95
TDZ (3 μM) +NAA(1 μM)	^k^9.1±0.56	^g^3.1±0.15	97
TDZ (4 μM) +NAA(1 μM)	^l^10.4±0.43	^k^3.6±0.67	97
TDZ (5 μM) +NAA(1 μM)	^o^13.4±0.26	^m^4.0±0.26	100
TDZ (1 μM) +IAA(1 μM)	^i^8.4±0.54	^f^2.8±0.36	95
TDZ (4 μM) +IAA(1.5 μM)	^l^10.5±0.26	^f^2.9±0.36	97
TDZ (8 μM) +IAA(2 μM)	^m^11.5±0.57	^f^2.9±0.37	98
TDZ (10μM)+IAA(2.5 μM)	^n^12.0±0.37	^g^3.0±0.46	100
TDZ (12 μM) +IAA(3 μM)	^n^12.9±0.45	^i^3.3±0.62	100
TDZ (14μM)+IAA(3.5 μM)	^n^12.9±0.42	^i^3.3±0.24	100
TDZ (1 μM) +IBA(1 μM)	^j^8.9±0.36	^h^3.2±0.16	96
TDZ (4 μM) +IBA(1.5 μM)	^mn^11.9±0.56	^m^3.9±0.65	100
TDZ (8 μM) +IBA(2 μM)	^n^12.7±0.15	^m^3.9±0.34	100
TDZ (10μM) +IBA(2.5 μM)	^o^13.6±0.35	^m^4.0±0.26	100
TDZ (12 μM) +IBA(3 μM)	^o^13.8±0.25	^m^4.0±0.46	100
TDZ (14μM) +IBA(3.5 μM)	^p^14.4±0.26	^m^4.0±0.67	100
TDZ (16 μM) +IBA(4 μM)	^o^13.4±0.45	^m^4.0±.67	100
TDZ (20μM) +IBA(4.5 μM)	^o^13.0±0.56	^l^3.8±0.29	100

*Values are represented as mean±SD (n = 10), Data was analyzed by ANOVA using Duncan’s multiple range test (SPSS17.0); the values with different superscript along the columns are statically significant at P<0.05. Data scored after 8 weeks of the culture period.

Callus explants (without shoots) were taken for root development. Murashige and Skoog media augmented with auxins at different concentrations (IBA/IAA/NAA) was used for root development. A 100% direct rooting response was noticed with an average number of roots (8.5±0.68) and 4 cm of average root length (Table 5, Fig 2E) with IBA (8μM). Lower concentration of IAA and NAA favored an increase in root number. However, it was observed that NAA showed less effect on root formation. The order of effectiveness in terms of root induction was IBA>IAA>NAA.

**Table 5:** Effect of MS medium supplied with different concentrations of IBA/IAA/NAA on root induction from *in vitro* raised callus of *H*. *niger* L.

Treatments	Root Number	Root Length(cm)	Rooting (%)
Control (MS basal)	^a^1.1±0.45	^a^1.5±0.59	45
NAA(0.5μM)	^cd^2.3±0.28	^c^1.9±0.37	82
NAA(1.0μM)	^e^2.7±0.56	^b^1.7±0.85	85
NAA(2.0μM)	^d^2.4±0.79	^a^1.5±0.36	72
NAA(4.0μM)	^c^2.3±0.67	^b^1.7±0.88	69
NAA(8.0μM)	^b^2.2±0.89	^a^1.5±0.56	57
NAA(10.0μM)	^b^2.0±0.56	^g^2.4±0.37	55
IBA(0.5μM)	^e^2.6±0.48	^g^2.5±0.45	90
IBA(1.0 μM)	^j^4.9±0.38	^g^2.6±0.87	100
IBA(2.0μM)	^k^5.5±0.76	^ij^3.1±0.67	100
IBA(4.0μM)	^m^6.9±0.87	^j^3.3±0.79	100
IBA(8.0μM)	^n^8.5±0.68	^k^4.0±0.46	100
IBA(10.0μM)	^i^4.6±0.52	^hi^3.0±0.46	100
IBA(12.0μM)	^f^3.2±0.52	^hi^3.0±0.63	100
IAA (0.5μM)	^gh^3.6±0.35	^h^2.9±0.36	100
IAA (1.0μM)	^g^3.5±0.56	^f^2.3±0.52	83
IAA(2.0μM)	^h^3.8±0.42	^g^2.6±0.54	91
IAA(4.0μM)	^e^2.6±0.75	^ef^2.2±0.56	78
IAA (8.0μM)	^bc^2.2±0.57	^ef^2.2±0.33	72
IAA (10.0μM)	^b^2.0±0.51	^d^2.1±0.30	70

* Values are represented as mean±SD (n = 10), Data was analyzed by ANOVA using Duncan’s multiple range test (SPSS17.0); the values with different superscript along the columns are statically significant at P<0.05. Data scored after 8 weeks of culture period.

At least, 30 rooted plantlets were acclimated first in the lab and then transferred to greenhouse conditions out of which 26 plants i.e 86.6% survival was recorded after 3 months.

### Effect of Ethyl Methane Sulfonate on shoot and root regeneration and callus biomass

**Fig 3:**
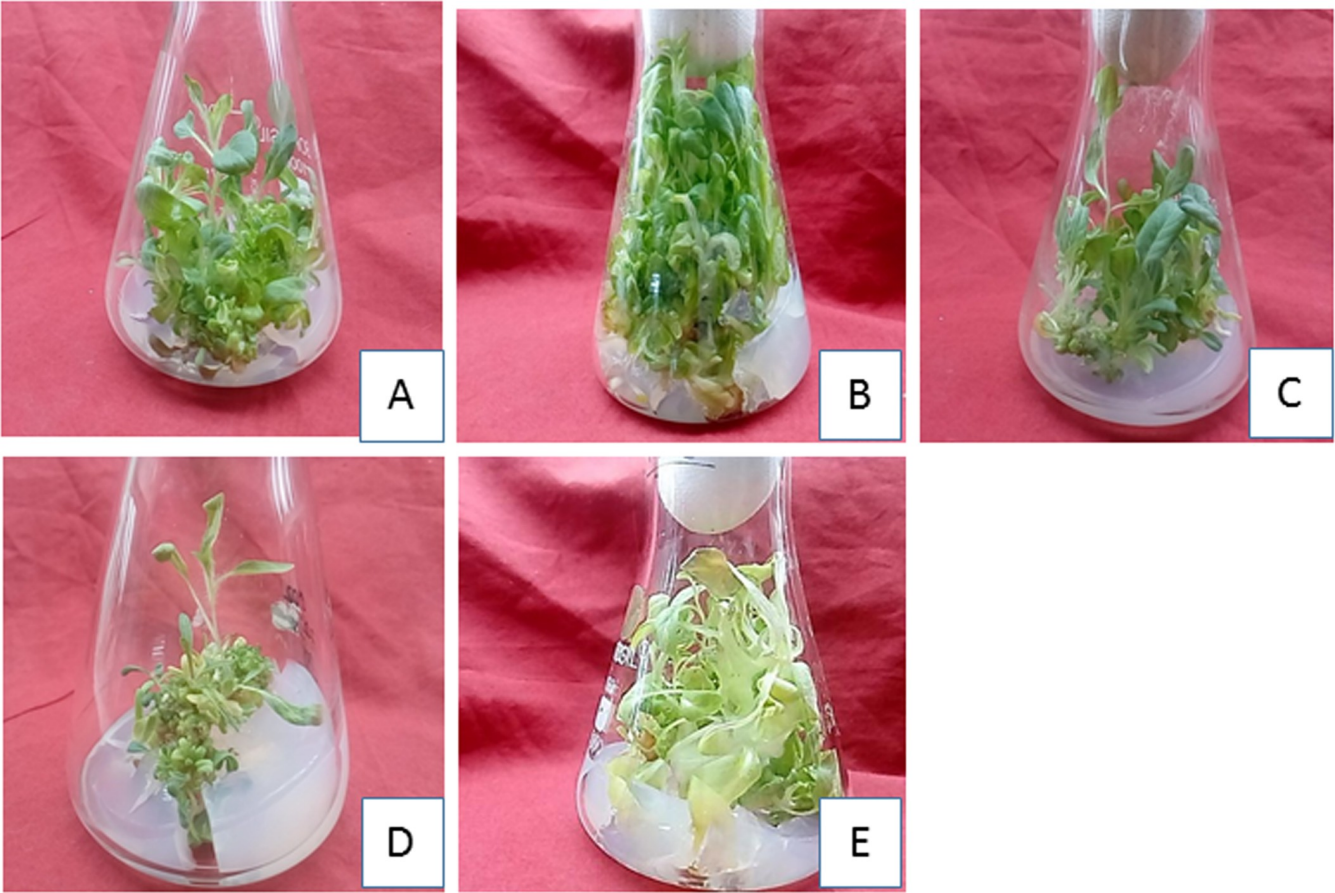
Effect of EMS on in vitro regeneration potential of callus of H. niger on MS +TDZ (14μM)+IBA(3.5 μM). (A) Control (0%), (B) 0.03% EMS, (C) 0.05% EMS, (D) 0.08% EMS, (E) 0.1% EMS.

**Fig 4:**
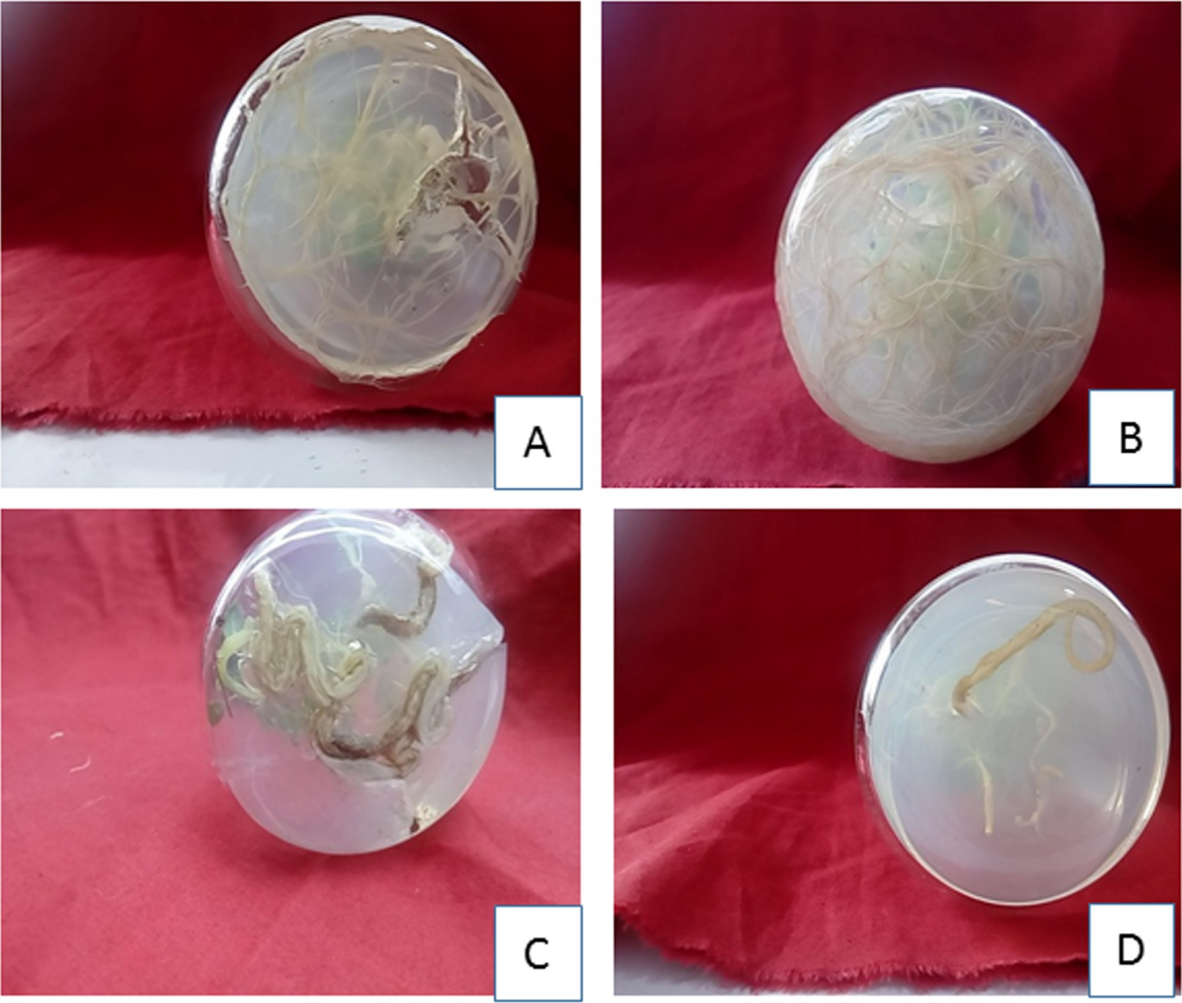
Effect of EMS on rooting from *in vitro* raised callus of *H*. *niger* L. (A) Control (0%),(B) 0.03% EMS, (C) 0.05% EMS, (D) 0.1% EMS.

**Fig 5:**
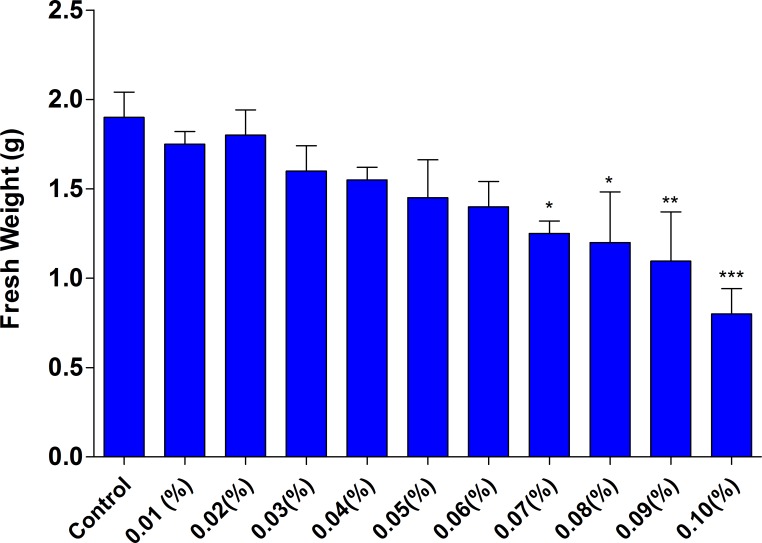
Effect of EMS on callus biomass (fresh weight) from *in vitro* raised callus of *H*. *niger*. L. The samples were collected after 6 weeks of incubation period. Data indicates the mean of three biological replicates. For each replicate, a total of 10 explants were used. An asterisk represents the significant differences between explants treated with EMS compared to control **P<0.05; **P<0.01; ***P<0.001.

Callus obtained from Kn (1.5 μM)+NAA(12μM) was treated with different concentration of EMS (0.01% -0.1%) for1 hour and cultured on MS +TDZ (14 μM) + IBA(3.5 μM) and MS+IBA (8 μM) for shoot and root regeneration respectively. EMS showed an intense effect on shoot and root regeneration potential as shoot number and root number increased as compared to control upto threshold level. The shoot number (22.2 ±0.77) and root number (12.8±0.53) showed increasing trend up to 0.03% EMS. However, with increase in concentration of EMS the shoot number and root number showed decrease trend (Tables 6 and 7, Figs 3 and 4). Lethal dose was recorded at 0.08% EMS where 50% of the population was dead as compared to control. However, the shoot length and root length (Tables 6 and 7) and callus biomass (Fig 5) showed gradual decrease with increase in EMS concentration.

**Table 6:** Effect of Ethyl Methane Sulfonate on shoot regeneration from *in vitro* raised callus of *H*. *niger* L.

Time Duration (Hour)	Treatments EMS (% v/v)	Shoot No	Shoot length (cm)	Shooting (%)
Control	0%	^e^14.5±0.30	^c^4.0±0.15	100
1-hour	0.01	^ef^15.0±0.79 (4.1%)	^c^3.7±0.59 (-7.5%)	95
	0.02	^g^20.2±0.69 (40.2%)	^b^3.7±0.56(-7.5%)	100
	0.03	^h^22.2 ±0.77 (81.9%)	^b^3.4±0.53 (-15%)	100
	0.04	^g^19.8 ±0.42 (37.5%)	^b^3.1±0.81 (-22.5%)	100
	0.05	^e^13.6 ±0.43 (-5.5%)	^ab^3.1±0.t3 (-22.5%)	100
	0.06	^a^12.8±0.23 (-11.7%)	^ab^3.1±0.36 (-22.5%)	100
	0.07	^d^11.2±0.44 (-22.2%)	^a^2.5±0.59 (-37.5%)	95
	0.08	^c^7.2±0.45 (-50.0%)	^a^2.0±0.56 (-50.0%)	80
	0.09	^b^4.1±0.67 (-71.50%)	^a^1.9±0.30 (-52.5%)	80
	0.1	^a^2.0±0.53 (-86.2%)	^a^1.5±0.59 (-62.5%)	70

* MS media was supplemented with using TDZ (14 μM) +IBA (3.5μM) *in vitro* raised callus as explants. Values are represented as mean±SD (n = 10), Data was analyzed by ANOVA using Duncan’s multiple range test (SPSS17.0); the values with different superscript along the columns are statically significant at P<0.05. Data scored after 8 weeks of culture period. ** Percent variation is mentioned in each cell below the main value along the column.

**Table 7:** Effect of Ethyl Methane Sulfonate on rooting from *in vitro* raised callus of *H*. *niger* L.

Time (Hour)	Treatments EMS (% v/v)	No. of roots	Root length (cm)	Rooting (%)
Control	0%	^e^8.5±0.31	^g^4.0±0.11	100
1 hour	0.01	^e^8.8±0.11 (3.5%)	^f^3.7±0.34 (-7.5%)	100
	0.02	^f^10.2±0.23 (20%)	^f^3.7±0.22 (-7.5%)	100
	0.03	^h^12.8±0.53 (50.5%)	^e^3.3±0.33 (-17.5%)	100
	0.04	^g^11.2±0.41 (32.9%)	^e^3.3±0.24 (-17.5%)	100
	0.05	^f^10.1±0.43 (18.8%)	^d^3.1±0.24 (-22.5%)	100
	0.06	^e^8.6±0.34 (1.1%)	^d^3.0±0.13 (-25%)	100
	0.07	^d^7.7±0.42 (-9.4%)	^c^2.8±0.45 (-30%)	100
	0.08	^c^4.2±0.23 (-50.5%)	^b^2.0±0.26 (-50%)	85
	0.09	^b^2.1±0.66 (-75.2%)	^a^1.5±0.25 (-62.5%)	80
	0.1	^a^1.1±0.12 (-87%)	^a^1.5±0.31 (-62.5%)	70

* MS media was supplemented with IBA (8 μM). Values are represented as mean±SD (n = 10), Data was analyzed by ANOVA using Duncan’s multiple range test (SPSS17.0); the values with different superscript along the columns are statically significant at P<0.05. Data scored after 8 weeks of culture period. ** Percent variation is mentioned in each cell below the main value along the column.

### Mutational analysis in PMT and H6H gene

**Fig 6:**
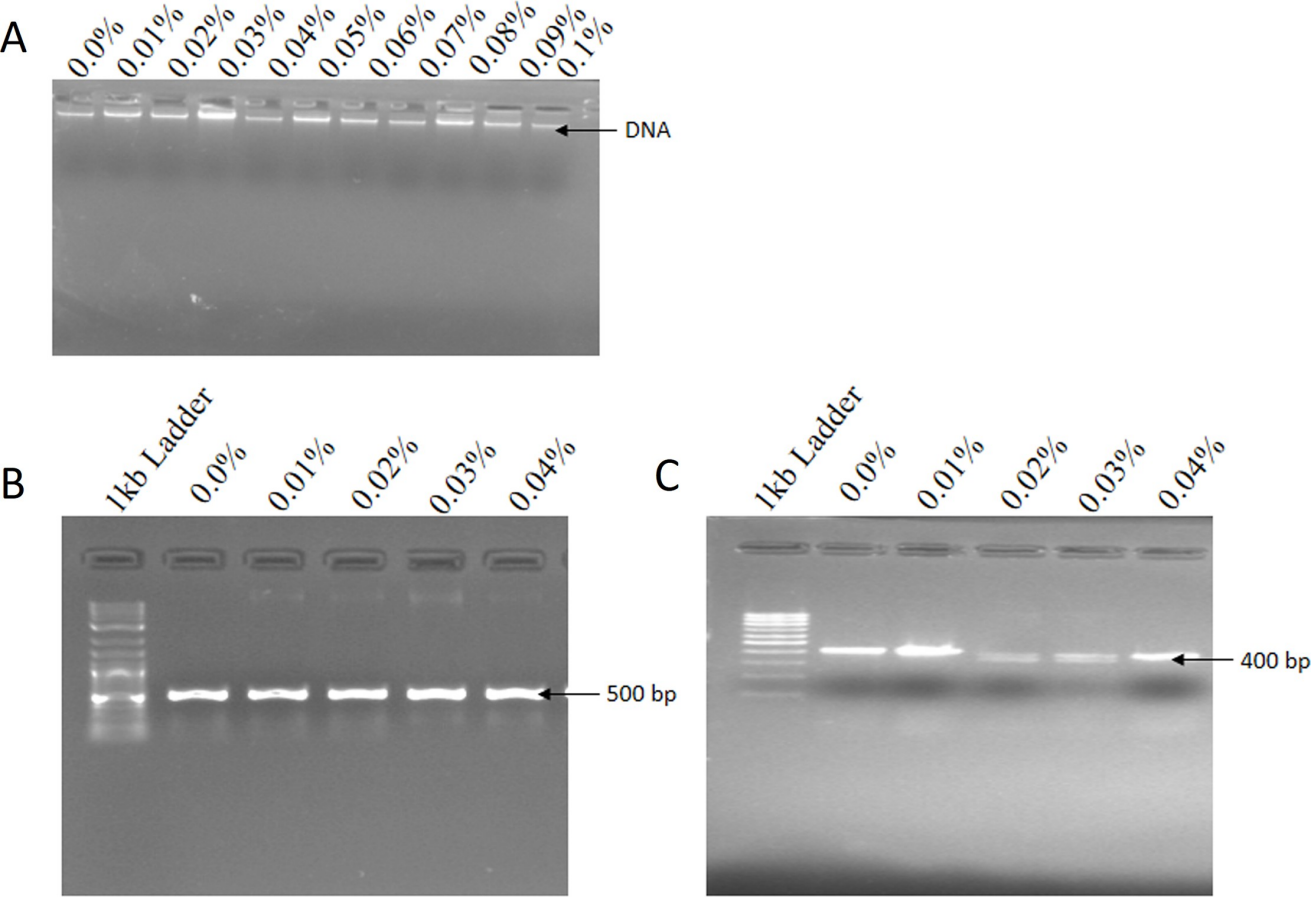
Representative image of DNA extraction and PCR analysis of H6H and PMT gene isolated from EMS treated and control explants of *H*. *niger* of different samples (500 and 400bp respectively) of (A) DNA extraction isolated from explants treated with different concentrations of EMS and untreated. (B) Depicts the PCR analysis of H6H (500bp) Gene (C) PCR amplification PMT Gene (400bp).

**Fig 7:**
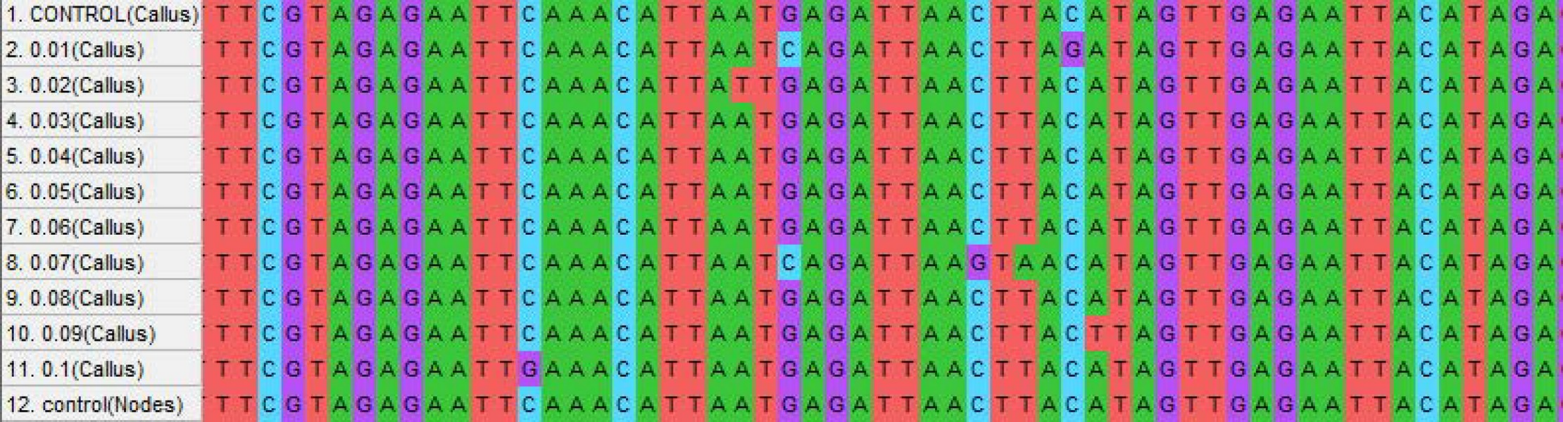
Multiple sequence alignment of samples generated from PMT gene amplification showing various base pair changes (shown by colour change). The fasta sequences of samples were aligned with the reference sequence of PMT gene available from NCBI website using MEGA-X software.

**Fig 8:**
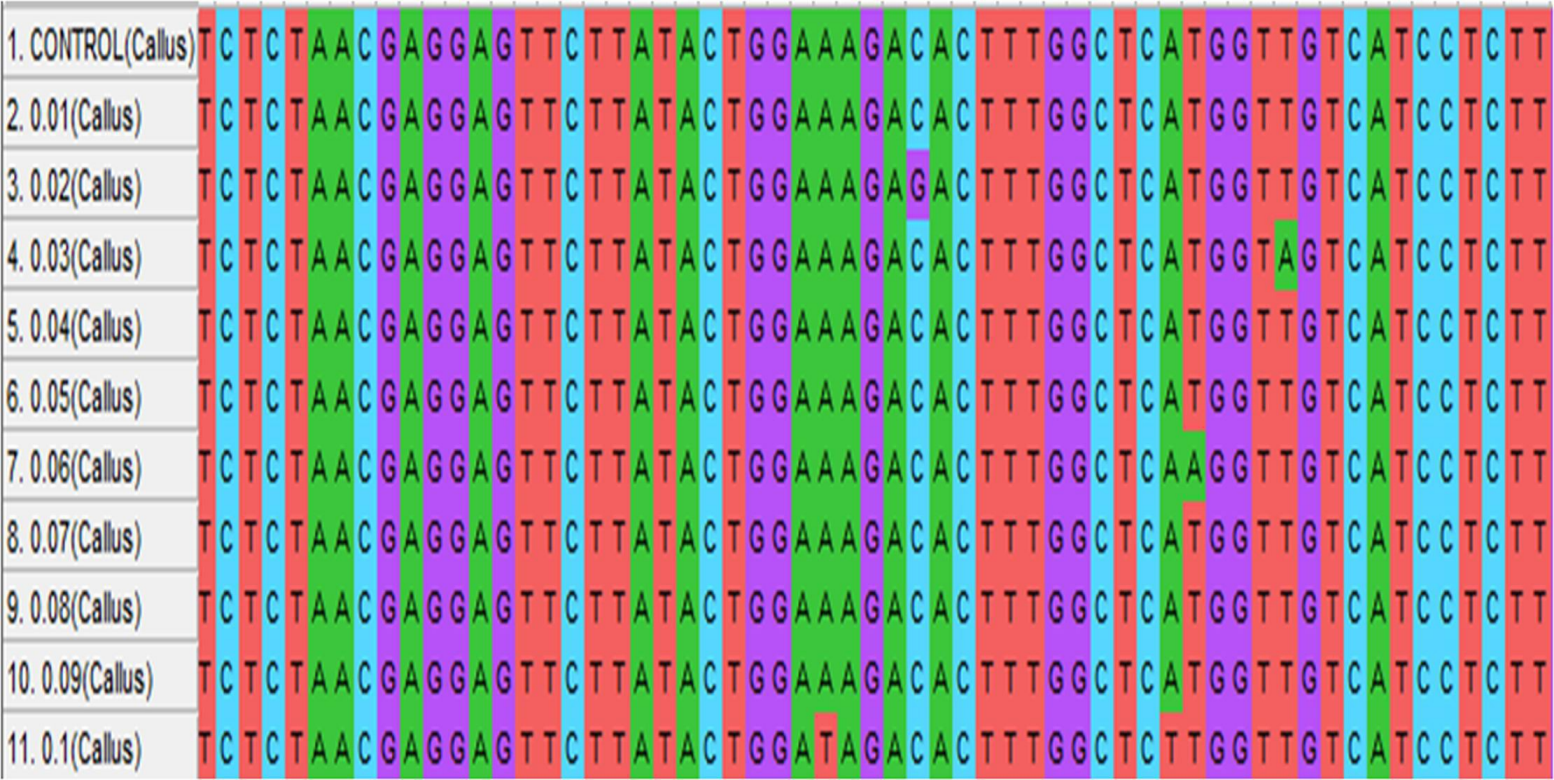
Multiple sequence alignment of samples generated from H6H gene amplification showing various base pair changes (shown by color change). The fasta sequences of samples were aligned with the reference sequence of H6H gene available from NCBI website using MEGA-X software.

An effective DNA isolation protocol from leaf samples was obtained in the present study (Fig 6A). Gene sequences available at National Center for Biotechnology Information (NCBI) (www.ncbi.nih.gov) were used for primer designing by Gene Runner Software (Hasting Software, Inc., Hasting, NY). The primers were synthesized by Eurofins Genomics (Bangalore India). DNA isolated from treated and control plants were used as a template for PCR (Polymerase chain reaction) targeting H6H and PMT gene for amplification (Fig 6B and 6C). The result obtained from sequencing was analyzed using MEGA-X software. The fasta sequence of the mutant plants was aligned with the reference sequence of PMT and H6H gene available from NCBI website. The Screening of Single Nucleotide Polymorphism (SNPs) shows mutations in terms of G/C, A/T and G/A (Table 8, Figs 7 and 8). However, no nucleotide variation was observed in control (untreated). The variations in full-length sequences of PMT and H6H genes amplified from control and EMS treated samples are given in S1 Fig.

**Table 8:** Nucleotide variations in PMT and H6H genes following different Ethyl Methane Sulfonate concentrations.

Treatments EMS (% v/v)	PMT gene		H6H gene	
	Wild Type	Mutant Type	Wild Type	Mutant Type
Control	No Change		No Change	
0.01	GC	CG	A	T
0.02	A	T	C	G
0.03	TGG	ACC	TA	AT
0.04	TTG	AAC	CT	GA
0.05	GGT	CCA	TA	AT
0.06	ATG	TAC	T	A
0.07	GCT	CGA	No Change	
0.08	No Change		TG	AC
0.09	A	T	GT	CA
0.1	C	G	AA	TT

Alphabets here represent A (Adenine); G (Guanine); T (Thymine); C (Cytosine).

### Transcript levels of PMT and H6H on exposure to different concentrations of EMS

**Fig 9:**
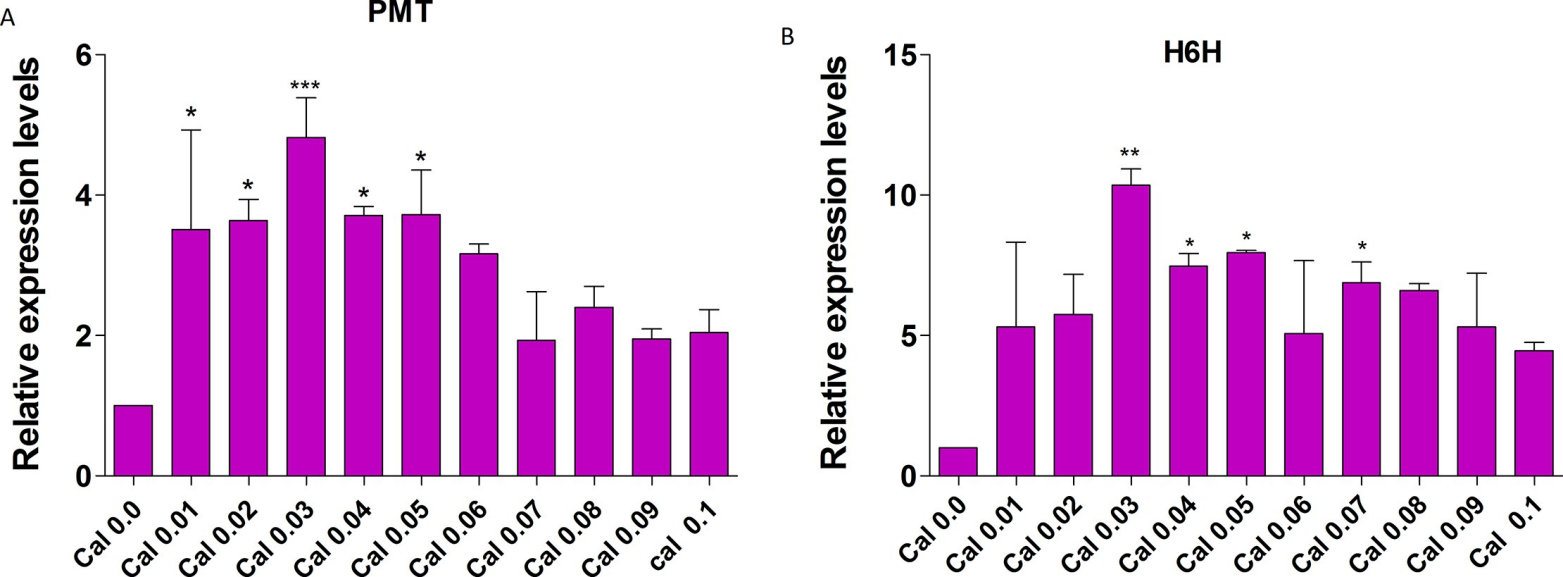
Transcript expression analysis of PMT and H6H in *Hyoscyamus niger* callus treated with different concentrations of EMS mutagen. (A) Expression analysis of PMT (B) expression analysis of H6H. The column represents the mean of three biological repeats. Asterisks represent the statistically significant differences between EMS treated explants and control (untreated) at *P<0.05; **P<0.01; **P<0.001.

Since the morphological data revealed an increase in the morphological characteristics after EMS treatment at low concentrations, it entailed a need to elucidate the molecular mechanisms of EMS mutation by evaluating the expression of two key genes (PMT and H6H) that are pivotal in the synthesis of secondary metabolites. It was clear from the results obtained from qPCR that there was an increase in expression of PMT and H6H genes at different concentrations of EMS starting from 0.01% to 0.03%. However, from 0.04% to 0.1% we observed a gradual decline in the expression of these genes. At 0.03% EMS H6H was found to be increased by 10.3 fold concerning its respective control (Fig 9). The expression of PMT was also found to be higher in samples treated with 0.03% EMS with a 4.8 fold increase compared to the untreated control sample (Fig 9). The results we obtained suggested an increase in the accumulation of H6H and PMT mRNA transcripts in response to EMS treatment relative to the untreated control, corroborates with our HPLC data that showed a spike in the expression of secondary metabolites in treated samples with optimal at 0.03% EMS.

### Quantitative analysis

**Fig 10:**
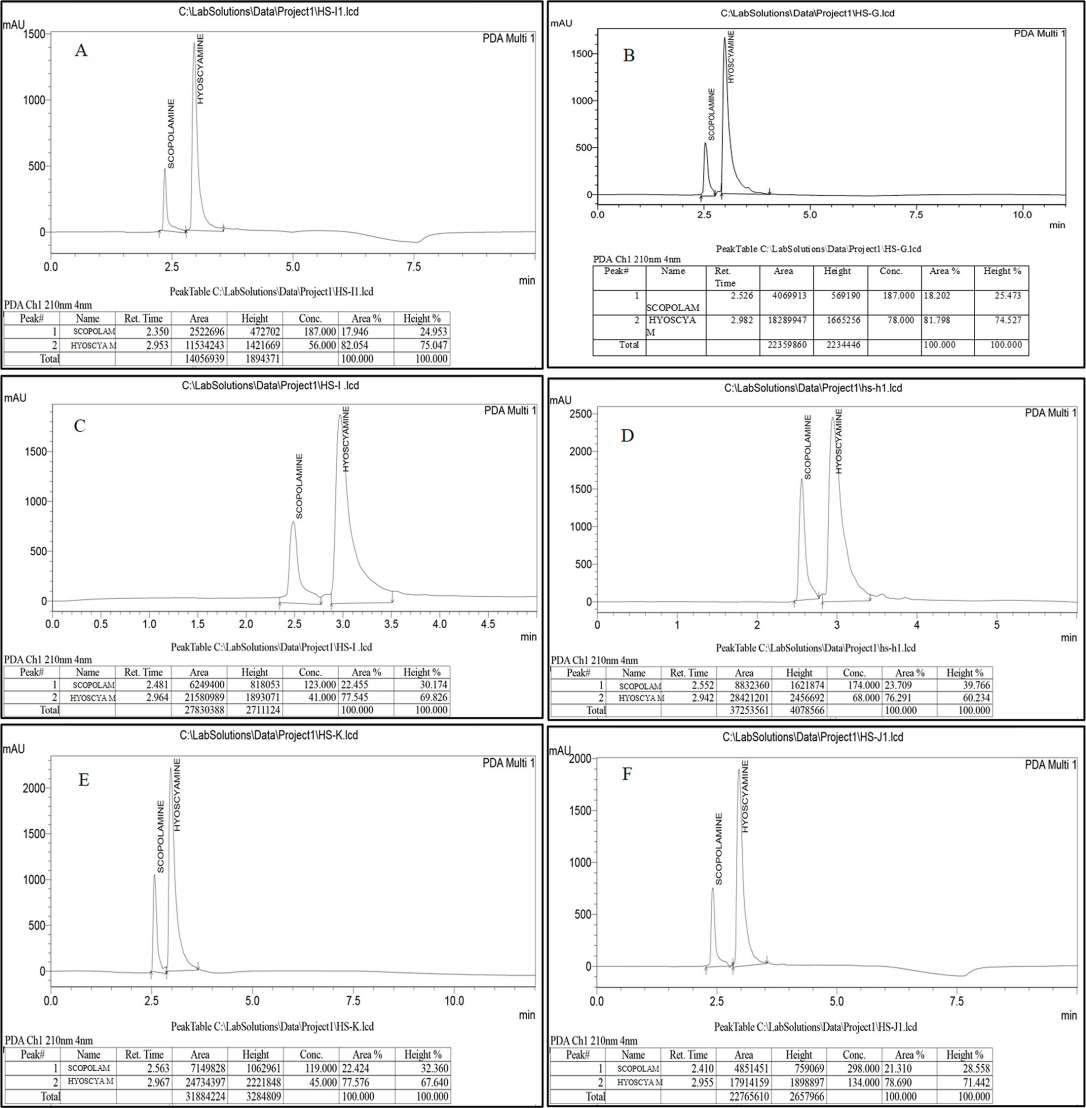
HPLC analysis of hyoscyamine and scopolamine extracted from callus treated with different concentrations of EMS. (A-F) representative peaks of hyoscyamine and scopolamine content in callus treated with 0.0%, 0.01%, 0.02%, 0.03%, 0.04 and 0.05% EMS, respectively.

HPLC analysis of hyoscyamine and scopolamine was performed in EMS treated samples ranging from (0.01–0.05) and respective control, which were selected based on data from expressional analysis. The quantitative analysis revealed that variants developed at 0.03% EMS accumulated the highest amount of scopolamine (0.0639μg/g) and hyoscyamine (0.0344μg/g) relative to control which had 0.0139μg/g of hyoscyamine and 0.0182μg/g of scopolamine (Table 9, Fig 10). While minimum concentration of both hyoscyamine (0.0271μg /g) and scopolamine (0.0351μg/g) was recorded at 0.05% EMS (Table 9, Fig 10). Scopolamine was found to be the predominant tropane alkaloid as compared to hyoscyamine.

**Table 9:** Variation in hyoscyamine and scopolamine content in *Hyoscyamus niger* following exposure to different concentrations Ethyl Methane Sulfonate.

Time (Hour)	Treatments EMS (% v/v)	Hyoscyamine Concentration (μg/g)	Scopolamine Concentration (μg/g)
1-hour	0% (Control)	a0.0139±0.003	a0.0182±0.001
	0.01	b0.0221±0.001	b0.0294±0.007
	0.02	c0.0261±0.004	d0.0452±0.007
	0.03	f0.0344±0.005	f0.0639±0.005
	0.04	e0.0300±0.001	e0.0518±0.002
	0.05	d0.0271±0.002	c0.0351±0.003

Values are represented as mean±SD (n = 3), Data was analyzed by One-Way ANOVA using Duncan’s multiple range test (SPSS17.0); the values with different superscript along the columns are statically significant at P<0.05.

## Discussion

Induced mutation has become a potent method for the development of new and novel germplasm [27]. It provides raw materials for the genetic improvement of commercially essential plants and also used to create genetic variability in a short time in quantitative and qualitative traits [28]. Therefore, the study was carried out for the genetic improvement of *Hyoscyamus niger* and may prove helpful for the stimulation of secondary metabolites.

Sterilization of explants is necessary before exposing it for *in vitro* propagation as it confirms the decrease in contamination rate and an increase in the survival rate of plantlets. In present study, 100% sterilization and 100% survival rate was obtained at 0.02% (w/v) HgCl_2_ for 25 minute. Chlorine being electronegative oxidizes the peptide linkages and therefore denatures microbial protein. HgCl_2_ is most commonly used for effective sterilization in various plants like *Berginia ciliate* [29], *Cichorium inlybus* [30], *Catharanthus roseus* [31]. Many researchers have used HgCl_2_ for surface sterilization [32, 33] under *in vitro* conditions. In contrast, the use of sodium hypochlorite for surface sterilization of explants has also been widely reported [34, 35] however, in this study NaClO proved less effective in the sterilization of explants, resulting in a brownish color, high contamination rate with less explant survival.

Callus is an unorganized mass of cells that develop when cells are wounded and play an important for plant cell cultures [36]. The high callus formation in the present study [Kn (1.5 μM) +NAA (12μM)] may be due to the high auxin and low cytokinin concentration, that promotes cell proliferation [37]. The results obtained are in agreement with previous reports in *Hyoscyamus muticus*, where the highest callus formation was found on MS media with 2 and 1 mg/L NAA and 0.5 mg/L kinetin [17]. In *Ipomoea obscura* similar results were obtained from leaf explants using 2,4-D+Kn [38]. Leaves were also used for the callus initiation in *Stevia rebaudiana* where the individual concentration of 2, 4-D, NAA, and BAP were used along with the MS basal media [39]. In this study, maximum callus biomass (1.8g/explant fresh weight) was obtained with Kn + NAA. Similar results were observed in *Celosia argentea* and Stevia *rebaudiana* that showed the highest biomass of 0.11 g/L and 2.0mg/L using leaf explants [40, 41]. Best growth and maximum biomass of callus was acquired on MS (BAP+NAA) through leaf explants by in *Hyoscyamus aureus* [42].

Regeneration of plantlets by cell, tissue, and organ culture is a major goal of plant tissue culture and shoot formation is desirable and a necessity for every tissue culture protocol. The study revealed that MS+ TDZ (14μM)+ IBA (3.5μM) showed a maximum number of shoots from callus explants and TDZ proved to be the best hormone for shoot proliferation. Further, TDZ has been considered to be more powerful and the most commonly used cytokinins [43] to stimulate shoot regeneration in plants [44]. Our findings are in confirmation with several workers in various plants like *Sorghum bicolor*, rice, *Arnica Montana* [45–47] showed the best shooting response from callus on MS medium augmented TDZ.

In the present study, lower doses of EMS were found to be stimulatory for different morphogenetic responses and higher doses showed an inhibitory effect. This stimulatory effect was observed at lower concentrations of EMS may be due to the increase in cell division rates plus activation of growth hormones e.g., auxin and cytokinins [48]. However, lower doses could also induce physiological and biochemical changes [49, 50], resulting in faster vegetative growth, instead at higher doses, it inhibits cell multiplication [51]. Growth promoting effects of mutagens when applied at low dozes have earlier been recorded in various crop plants such as potato [52], *Picrohiza kurroa* [53] and tomato [54]. The variation at high concentration of EMS may be due to the toxicity [55], the genotoxic effect that arrests the cell cycle or chromosomal aberrations [56]. Similar results were obtained in *Coriandrum sativum* L. where the mutagenic effect of EMS was recorded on biochemical components, cytological features, and morphology. The results showed negative effects with a higher concentration of EMS for a longer duration compared to lower concentrations of EMS [57]. Furthermore, a gradual decline in shoot length with an increase in gamma radiation frequency under *in vitro* conditions was recorded in *Gyprophila paniculata* [58].

The induction of chemical mutation in combination with molecular methods has proved effective in creating variation in terms of nucleotide substitution variation in genes (PMT and H6H) in *Hyoscyamus niger*. EMS is commonly used in plant systems as a chemical mutagen for DNA lesions by inducing base changes or nucleotide substitution, consequently alter codon sequences, leading to either non-synonymous or synonymous effects[10]. The point mutations caused by EMS are single-base substitutions that may arise due to transitions (purine to purine or pyrimidine to pyrimidine) and transversions (pyrimidine to a purine) [59]. These variations in the genes are the precursors of the secondary metabolites that lead to an increase in the content of hyoscyamine and scopolamine, as lower concentrations of EMShad a stimulatory effect which was evident through morphological characters.

**Fig 11:**
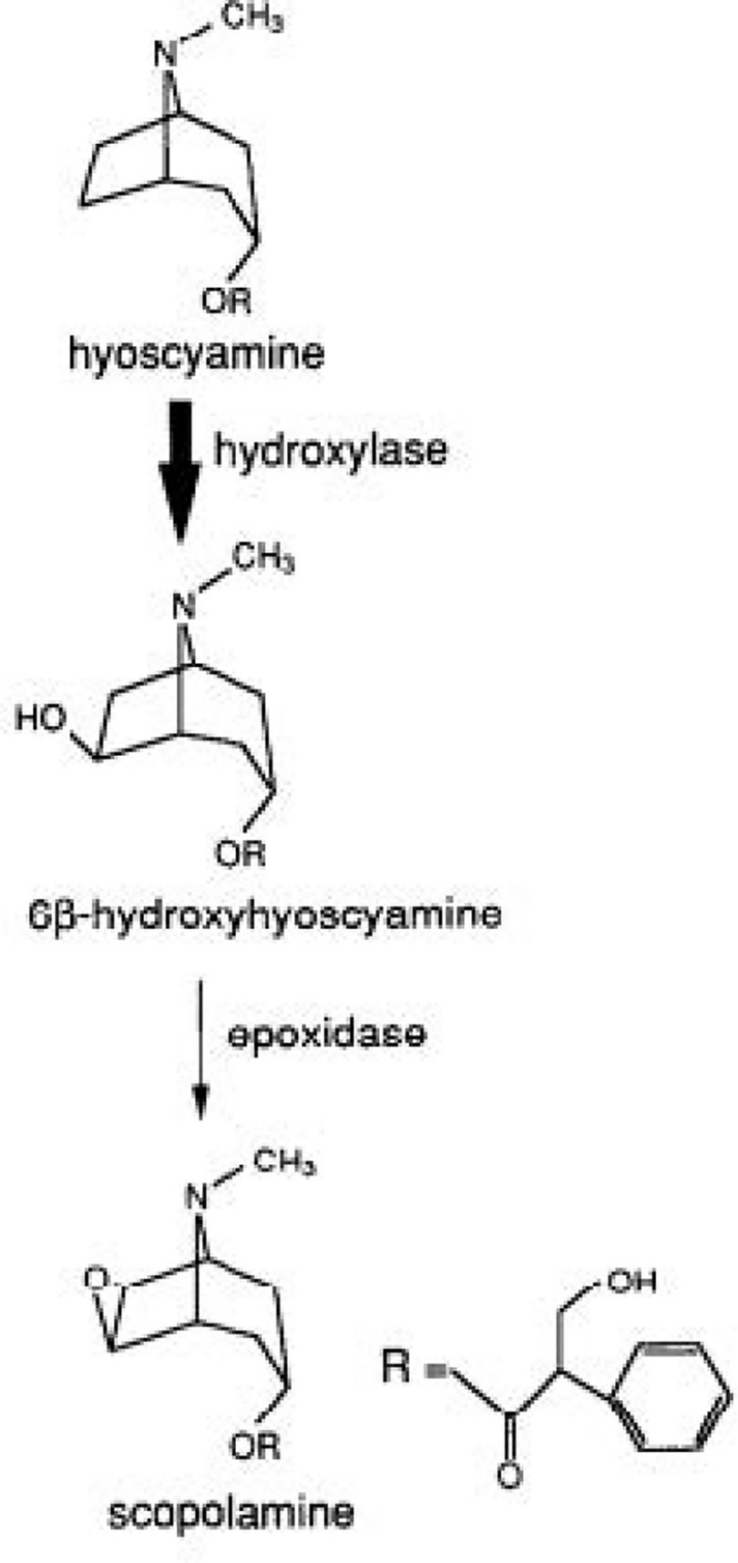
Conversion of hyoscyamine to scopolamine. Schematic representation of scopolamine production from hyosyamine.

Plant alkaloids constitute the major group of natural compounds, providing numerous pharmacologically active products that are synthesized as secondary metabolites [60]. Production and isolation of these secondary metabolites from plant tissue culture has emerged as a promising and viable option of research [61]. Since the first specific precursor of the tropane alkaloid pathway is N-methylputrescine, whose formation from putrescine is catalyzed by PMT and hence represents the first committed step in tropane alkaloid biosynthesis. And H6H encodes an enzyme catalyzing the final two steps from hyoscyamine to scopolamine, these two genes were selected for the induction of mutations using EMS (Fig 11). The present study has revealed that overexpression of H6H and PMT genes due to EMS mutation led to an increase in secondary metabolite content (hyoscyamine and scopolamine). Increase in the scopolamine and hyoscyamine concentration at 0.03% EMS in comparison to control has been recorded through HPLC analysis, which may be a result of overexpression of H6H and PMT genes at low concentration, however, with increase in EMS concentration there was a down-regulation of H6H and PMT genes that ultimately lead to decrease in the concentration of secondary metabolites. This downregulation in gene expression may be due to modification in enzyme activity or inhibition in the physiological activities due to EMS [62] or may be due to the high rate of nucleotide variations at higher concentrations of EMS. Therefore, It was also concluded that a decrease in quantitative and qualitative traits have been attributed to the physiological disturbance or chromosomal damage caused to the cells of the plant by the mutagen [63]. Upregulation in gene expression may be due to the induction of physiological and biochemical changes [50] and due to changes in hormonal signaling network of the cells [64]. Our results are in consensus with many researchers who reported the expressional analysis of PMT and H6H genes in transgenic plants and their upregulation increases in the hyoscyamine and scopolamine concentrations in *Hyoscyamus niger* [65], *Atropa belladonna* [66], S*copolia parviflora* [67].

Our study showed a maximum increase of 10.3 and 4.8 fold in the expression of H6H and PMT, respectively, at 0.03% EMS to control, suggesting that EMS at low concentration leads to an increase in expression, whereas the decrease in expression is seen with a higher concentration of EMS. There are reports related to the expression of PMT and H6H in other plant species; however, to our knowledge, this is the first study to evaluate the effect of EMS on gene expression of PMT and H6H and its effect on secondary metabolite concentration. Overexpression of PMT and H6H in *Hyoscyamus niger* and *Atropa belladonna* in transgenic hairy root cultures [65, 66] and expressional analysis of H6H gene from *Hyoscyamus niger* and *Hyoscyamus tenuicaulis* [68] has been reported. Our study is also in agreement with the work carried out regarding the expressional analysis of PMT and H6H genes in S*copolia parviflora* [67].

## Conclusion

Taken together, these results suggest a pivotal role of H6H and PMT genes in sustaining the tropane alkaloid pathway in *H*.*niger* and offers a valid reason for enhanced expression of these genes that could enhance the scopolamine and hyoscyamine content of the plant and hence improving the pharmacological properties of this plant. Moreover, the study was effective in creating variation in terms of phenotypic variation, nucleotide variation, overexpression of genes (PMT and H6H) and increase in secondary metabolite content of *Hyoscyamus niger* L. Thus the study may pave way for large-scale production of scopolamine and hyoscyamine by using EMS in *Hyoscyamus niger*. The protocol herein also described is an efficient and reproducible method for *in vitro* multiplication and *in vitro* mutagenesis of *H*. *niger* through indirect organogenesis.

## Supporting information

S1 Table (DOCX)

S2 Table (DOCX)

S1 Fig (DOCX)

S2 Fig (DOCX)
