# Supplementary material for: Promoting the accumulation of scopolamine and hyoscyamine in Hyoscyamus niger L. through EMS based mutagenesis
Source: PLoS One. 2020 May 21;15(5):e0231355. doi: 10.1371/journal.pone.0231355 (PMC7241962; doi:10.1371/journal.pone.0231355)
Supplement: S1 Table — (DOCX) [file pone.0231355.s001.docx]

**Table S1. Full-length PCR primer for PMT and H6H genes**

| Primer Name | Direction | Sequence (5’ 🡪 3’) | PCR product (bp) |
| --- | --- | --- | --- |
| PRIMER I  (PMT ) | Forward (SI) | GGCAACCACCATAAACAAGGTCTTTTAAC | 1475 |
|  | Reverse (RI) | CCGGCTCTTTTAAAAAAGAACAATTATTTAAAG |  |
| PRIMER II  (H6H) | Forward (SII) | ATGGCTACTTTTGTGTCGAACTG | 1035 |
|  | Reverse (RII) | TTAGACATTGATTTTATATGGCTTAACACC |  |
