# Supplementary material for: Promoting the accumulation of scopolamine and hyoscyamine in Hyoscyamus niger L. through EMS based mutagenesis
Source: PLoS One. 2020 May 21;15(5):e0231355. doi: 10.1371/journal.pone.0231355 (PMC7241962; doi:10.1371/journal.pone.0231355)
Supplement: S2 Table — (DOCX) [file pone.0231355.s002.docx]

**Table S2. qPCR primer characteristics of PMT and H6H genes used for PCR analysis**

| Primer Name | Direction | Sequence (5’ 🡪 3’) | PCR product (bp) |
| --- | --- | --- | --- |
| PRIMER I  (PMT ) | Forward (SI) | TTCGAGGCGGTGACTAAAGC | 206 |
|  | Reverse (RI) | TCTGGTCCCTCCGTAGAACA |  |
| PRIMER II  (H6H) | Forward (SII) | GGATGCTACCTGGATTGCTGT | 224 |
|  | Reverse (RII) | AGAGTGGTGGGTTGTCTTGG |  |
