# Supplementary material for: Promoting the accumulation of scopolamine and hyoscyamine in Hyoscyamus niger L. through EMS based mutagenesis
Source: PLoS One. 2020 May 21;15(5):e0231355. doi: 10.1371/journal.pone.0231355 (PMC7241962; doi:10.1371/journal.pone.0231355)
Supplement: S1 Fig — (DOCX) [file pone.0231355.s003.docx]

**Fig. S1** Full-length sequencing results of H6H and PMT gene (Fasta format)
