# Supplementary material for: Promoting the accumulation of scopolamine and hyoscyamine in Hyoscyamus niger L. through EMS based mutagenesis
Source: PLoS One. 2020 May 21;15(5):e0231355. doi: 10.1371/journal.pone.0231355 (PMC7241962; doi:10.1371/journal.pone.0231355)
Supplement: S2 Fig — (DOCX) [file pone.0231355.s004.docx]

**
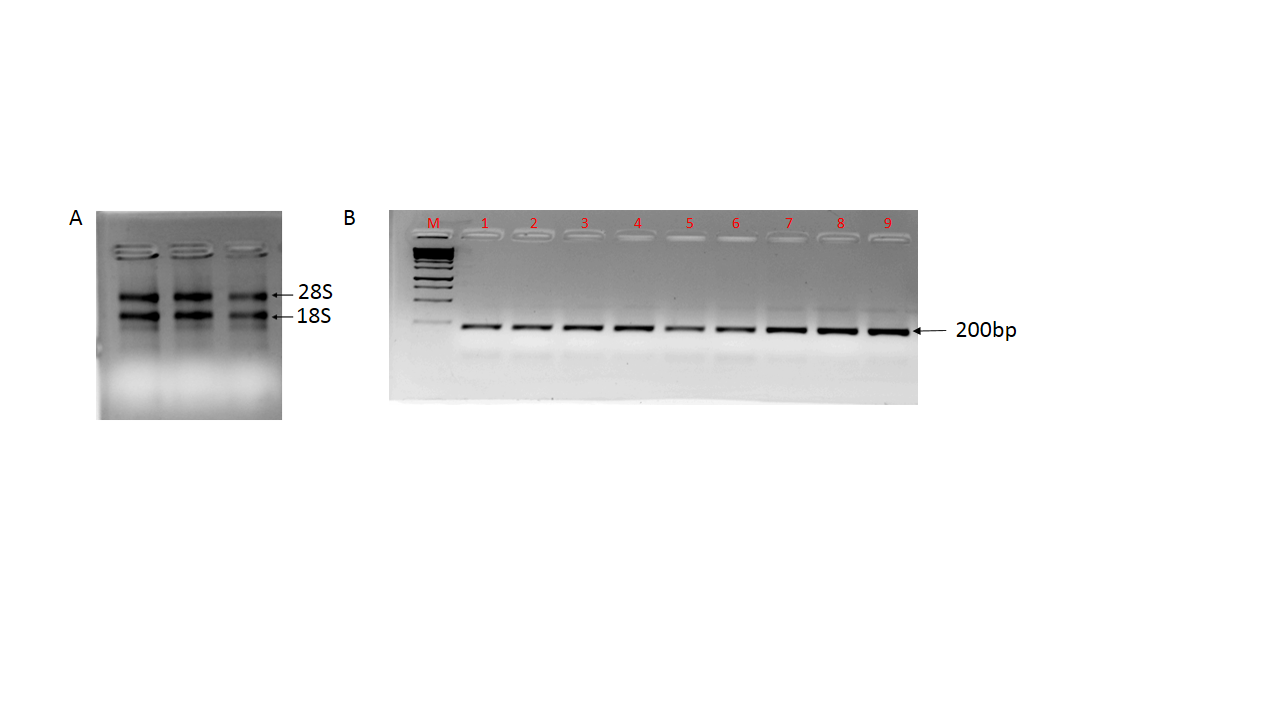
**

**Fig. S3 (A) representative of RNA extraction from different samples *H. niger* treated with EMS. Lane M images represent 1Kb DNA marker**. (B) represents the cDNA quality check isolated from different tissue samples using tubulin specific primers.
